# Mechanical Behavior of Multi-Phase Steels Comprising Retained Austenite

**DOI:** 10.3390/ma15020498

**Published:** 2022-01-10

**Authors:** Emin Semih Perdahcıoğlu, Hubert J. M. Geijselaers

**Affiliations:** Section of Applied Mechanics, Faculty of Engineering Technology, University of Twente, P.O. Box 217, 7500 AE Enschede, The Netherlands; h.j.m.geijselaers@utwente.nl

**Keywords:** martensitic phase transformation, DP steel, homogenization, self-consistent, formability, AHSS, transformation plasticity

## Abstract

The retained austenite (RA) in advanced high-strength steel (AHSS) grades, such as dual-phase (DP) steels, plays an important role on their formability. Thanks to the transformation-induced plasticity (TRIP) effect that occurs during the mechanically induced transformation of RA into martensite, additional ductility is obtained. Martensite has a higher flow stress than austenite; hence, the transformation results in an apparent hardening, which is beneficial for the stability of deformation. The stability of RA at a given temperature strongly depends on its carbon content, which, in AHSS, is not uniform but distributed. The aim of this study is to build a model that predicts the transformation as well as TRIP in a DP steel grade with RA. A physics-based kinetic model is presented that captures the transformation of retained austenite based on the thermodynamic driving force of the applied stress. A direct analytical estimate of transformation plasticity is provided, which is consistent with the kinetic model. Transformation kinetics is incorporated in a self-consistent, mean-field homogenization-based constitutive model. Finally, an indication of the effect of transformation of retained austenite on formability is given.

## 1. Introduction

Recent generations of advanced high-strength steel (AHSS) grades exploit complex microstructural effects such as stress and strain partitioning into various constituent phases in order to gain increased strength and formability. An important class of AHSS are dual-phase (DP) steels, which comprise ferrite and martensite phases with differing amounts for optimal performance. However, the behavior of DP steels can be further enhanced by allowing a certain amount of retained austenite (RA) phase to remain in the microstructure, resulting in those which are sometimes referred to as enhanced ductility dual-phase steels (DH). During deformation, it is observed that RA undergoes mechanically induced transformation into martensite, which gives rise to two beneficial effects. First, during transformation, an additional inelastic strain is obtained, which is referred to as transformation-induced plasticity (TRIP), which increases the ductility of the material. Moreover, second, the transformation into the harder martensite phase stabilizes the deformation, thereby increasing formability. As an extra benefit, DH steels retain the promising weldability of DP due to lower proportions of retained austenite and less alloying elements which influence weldability adversely.

The main challenge in modeling the mechanically induced transformation of retained austenite is identifying its driving mechanisms for which, in the literature, two different explanations can be found. The *strain-induced* transformation theory, proposed by Olson and Cohen [1], relates the amount of transformation to the formation of nucleation sites with the help of plastic deformation. Olson and Cohen [2] formulated a kinetic model which explains the martensite nucleation from ε-phase nucleation on shear band intersections during plastic deformation [3]. Stringfellow et al. [4] combined this kinetic model with mean-field homogenization in order to obtain the visco-plastic behavior of the material based on the constitutive behavior of the individual phases. Additionally, the model was extended to incorporate the influence of stress state and transformation plasticity. Further extensions have been added by Tomita and Iwamoto [5] for strain rate dependence and by Diani and Parks [6] for crystal plasticity. Stress dependence was added by Han et al. [7] through evaluating the mechanical driving force resolved on martensite variants; using this, the texture of the resulting martensite was determined. Beese and Mohr [8] built upon this model by considering Lode angle dependence. Papatriantafillou et al. [9] developed an elasto-plastic mean-field homogenization method using a strain-induced transformation model. Note that, in the strain-induced approach, the TRIP strain is an extra factor that needs to be described by an additional model and is often ignored in the above-mentioned studies. The Olson–Cohen model was successfully used by Serri et al. [10] in deep drawing simulations and by Lindgren et al. [11] for finite element simulations of hydro-forming. For simulations of the behavior of TRIP steel, the Olson–Cohen method has been widely used [12].

Tamura [13] proposed an alternative theory for mechanically induced martensite formation. In his model, only stress is considered to be driving the transformation. When the thermodynamic driving force, as formulated by Patel and Cohen [14], exceeds a threshold value, determined by chemical composition and temperature, the transformation starts. This has been theoretically and experimentally justified by Chatterjee and Bhadeshia [15], Das et al. [16] and He and Shang [17]. Furthermore, the strain-induced model has been shown experimentally to have problems in incorporating effects of prior plastic deformation on the transformation by Perdahcıoğlu et al. [18]. Due to the fact that the complete stress state is taken into account in this model, the dependency on hydrostatic stress, which manifests itself as tension–compression asymmetry, is inherent [19]. Several *stress-induced* transformation models have been developed, which are based on the thermodynamic action of the mechanical driving force, notably by Marketz and Fischer [20], Levitas [21] and Cherkaoui et al. [22]. In the stress-driven model, the transformation strain is inherently described as it is coupled with the driving force calculation as described in Perdahcıoğlu and Geijselaers [23]. Application has mainly been restricted to micro-mechanical simulations [24,25,26]. Hallberg et al. [27] and Perdahcıoğlu and Geijselaers [23] presented the application of the stress-induced transformation model in macroscopic scale for austenitic stainless steel and Lani et al. [28], Delannay et al. [29] and Kubler et al. [30] for TRIP steel.

In this research study, the goal is to model the overall mechanical behavior of AHSS with RA as one of the constituent phases. As these steels comprise multiple phases, a mean-field homogenization approach based on the self-consistent scheme was utilized in order to partition the strain and stress fields into each phase on an average level. This approach has been earlier utilized for two-phase mixtures by Perdahcıoğlu and Geijselaers [31]; however, it has also been generalized for more phases, as Lani et al. [28] presented a parallel Mori and Tanaka [32] model of both austenite and martensite in a ferritic matrix. Delannay et al. [29] formulated a hierarchic Mori–Tanaka model of martensite as inclusion in an austenite matrix and the aggregate of these as an inclusion in the ferritic–bainitic matrix. Kubler et al. [30] derived a self-consistent formulation with four separate phases. In that study, application of the mean-field method with tangent local stiffness and isotropic approximation of the strain polarization tensor, as formulated by Doghri and Friebel [33], was applied for more than two phases, a model which has been favorably reviewed by Chaboche et al. [34].

In the theory used in this paper, the transformation is modeled based upon the driving force resolved on martensitic variants residing in austenite grains with different orientations. The driving force is only correlated to the stress concentrated in austenite. As a consequence, once the stress and strain carried by austenite grains are determined via a mean-field model, the amount of transformation is determined using a defined relation between the maximum resolved driving force and the distribution of a critical energy barrier among the grains of RA based on grain orientation as well as carbon content.

Throughout the paper, following notations are used: 1st order tensors and vectors are shown in bold face and lowercase letters (a) 2nd order tensors and matrices in bold face and uppercase letters (A) and 4th order tensors with blackboard bold face and uppercase letters (A). $I^{\mathrm{vol}}=\frac{1}{3}1⊗1$ and $I^{\mathrm{dev}}=I-I^{\mathrm{vol}}$ denote the volumetric and deviatoric 4th order unit tensors, respectively. The single contraction of tensors is represented by a dot ($A\cdot b=c,A_{ij}\;b_{j}=c_{i}$), double contraction by a colon ($A:B=c,A_{ij}\;B_{ij}=c$) and fourth-order contraction by two colons, ($A::B=c,A_{ijkl}\;B_{ijkl}=c$). The dyadic (tensor) product is represented with the ⊗ sign (C=a⊗b, $C_{ij}=a_{i}\;b_{j}$).

## 2. Mean-Field Homogenization

The underlying principle of the mean-field homogenization method is to consider the average values of the field variables and their evolution in sub-domains, as well as their interactions. The overall (macroscopic) stress σ and strain ε are related to the average $\sigma_{i}$ and $\varepsilon_{i}$ in the individual phases by

(1) $$\sigma =\sum_{i}f_{i}\sigma_{i}\;\;\mathrm{and}\;\;\;\;\varepsilon =\sum_{i}f_{i}\varepsilon_{i}$$

The *f* stands for the volume fraction of each phase, which is subject to

(2) $$\sum_{i}f_{i}=1$$

In this paper, $i=\left(1,..,4\right)=\left(\gamma ,\alpha_{0}^{\prime },\alpha ,\alpha^{\prime }\right)$= (*austenite*, (*original martensite* or *bainite*), *ferrite*, *transformed martensite*). The transformed martensite inherits the local chemical composition within its parent austenite grain; therefore, its properties are different from the original martensite that has formed during production heat treatment.

As one of the main assumptions in this approach, the macroscopic constitutive relation defined for an individual phase is considered to be valid in the definition of the average stress–average strain relation for that phase within the compound.

(3) $${\overset{\circ }{\sigma }}_{i}=C_{i}:D_{i}$$

where $\overset{\circ }{\sigma }$ is an objective stress rate usually determined by finite element software, $D_{i}$ is the average rate of deformation in the $i^{\mathrm{th}}$ phase and $C_{i}$ is the elasto-plastic tangent determined by the constitutive law and the stress update algorithm.

Finally, strain concentration tensors $A_{i}$ are specified for each individual phase that provide the relation between the average rate of deformation in each phase $D_{i}$ and the overall rate of deformation D.

(4) $$D_{i}=A_{i}:D$$

which, by virtue of Equation (1), are subject to

(5) $$\sum_{i}f_{i}A_{i}=I$$

The homogenized response of the composite material is then found, in terms of the elasto-plastic tangent, as

(6) $$C=\sum_{i}f_{i}C_{i}:A_{i}$$

Material models that can be specified for the individual phases are not limited to isotropic hardening $J_{2}$-plasticity. For example, Doghri and Friebel [33] use a Chaboche [35]-type kinematic hardening model to capture the Bauschinger effect. On the other hand, Perdahcıoğlu and Geijselaers [31] show that, even with isotropic hardening specified for the phases, a Bauschinger effect shows up in the overall stress strain behavior.

Different homogenization schemes have been formulated using specific definitions of A. For use with two phases, the Mori and Tanaka [32] scheme is probably the most popular mean-field homogenization scheme. In the case of multiple phases, the bounds, e.g., iso-strain (Voigt–Taylor) and iso-stress (Reuss–Sachs), can be thought of as the most common schemes that are employed due to their simplicity. In this work, the self-consistent method where strain concentration tensors are calculated based on the work of Eshelby [36] was used.

### Self-Consistent Model

The earliest application of the self-consistent scheme can be found in [37,38]. In these studies, the plastic response of polycrystals was computed by taking into account the interaction of the matrix and individual crystals using Eshelby’s equivalent inclusion theory. This was extended to use for general matrix-inclusion systems by Hill [39]. In this scheme, each individual phase was considered to be an inclusion in the matrix which has the homogenized mechanical behavior of all comprising phases. The strain concentration tensor for phase *i* is then defined as [40]

(7) $$A_{i}={\left(I+S:\left(C^{-1}:C_{i}-I\right)\right)}^{-1}$$

The Eshelby tensor, S, depends only on the stiffness of the matrix phase and the aspect ratio of the ellipsoid inclusions. However, the homogenized stiffness of the matrix, C, depends on the strain concentration tensors of individual phases, as given in Equation (6). Consequently, an iterative solution procedure is needed for the calculation of the A-tensors.

Eshelby’s equivalent inclusion theory has been formulated for isotropic elastic matrix constitutive behavior. For use with an elasto-plastic matrix, the Eshelby tensor can be evaluated numerically. In this work, we followed the proposition by Doghri and Ouaar [41] to substitute the anisotropic material model by an isotropic comparison material model through an isotropic projection.

(8) $$\begin{array}{cc} \tilde{C}= & 3\tilde{\kappa }I^{\mathrm{vol}}+2\tilde{\mu }I^{\mathrm{dev}}\\ 3\tilde{\kappa }=C::I^{\mathrm{vol}}\; & ,\;2\tilde{\mu }=\frac{1}{5}C::I^{\mathrm{dev}} \end{array}$$

The expression for the Eshelby tensor for spherical inclusions in terms of $\tilde{\kappa }$ and $\tilde{\mu }$ is [40]

(9) $$\tilde{S}=\frac{3\tilde{\kappa }}{3\tilde{\kappa }+4\tilde{\mu }}I^{\mathrm{vol}}+\frac{6}{5}\frac{\tilde{\kappa }+2\tilde{\mu }}{3\tilde{\kappa }+4\tilde{\mu }}I^{\mathrm{dev}}$$

Therefore, the strain concentration tensors become

(10) $$A_{i}={\left(I+\tilde{S}:\left(C^{-1}:C_{i}-I\right)\right)}^{-1}={\left(I-\tilde{S}:C^{-1}:\left(C-C_{i}\right)\right)}^{-1}$$

As in Doghri and Ouaar [41], only Eshelby’s tensor was computed with the modulus of the isotropic comparison material. All other computations were performed with the consistent anisotropic elastic–plastic moduli from Equation (3). This is in contrast to Chaboche et al. [34], who proposed to evaluate the polarization tensor P as $P=\tilde{S}:{\tilde{C}}^{-1}$ instead of $P=\tilde{S}:C^{-1}$. Another difference is that, in the current formulation, a self-consistent model was used, whereas Doghri and Ouaar [41], as well as Chaboche et al. [34], used a Mori and Tanaka [32] model.

For detailed modeling of the martensitic transformation, it can be claimed that each individual martensite plate should be modeled as a thin ellipsoid with a specific orientation [22]. On the other hand, care must be taken in the physical considerations of the mean-field approach, as the result is an averaged macroscopic behavior. When averaged, a randomly oriented distribution of platelets is expected to result in an isotropic behavior. This can be captured in a mean-field approach when the mean shape of the martensite phase is considered as a sphere. In the case there is a preferential orientation of platelets, an ellipsoid inclusion would be suitable.

## 3. Austenite Transformation Model

Patel and Cohen [14] formulated the concept of mechanical driving force for the case of martensitic transformation under stress during quenching. The transformation model that is presented below builds upon the same concept applied to the transformation of retained austenite. It is known that martensitic transformation takes place through a diffusionless change in crystal structure. According to Wechsler et al. [42] and Bowles and MacKenzie [43], starting from the postulate of an invariant plane (habit plane) as interface between the martensite and the parent austenite, the transformation results in the following deformation gradient:

(11) $$F^{\mathrm{tr}}=1+m⊗n$$

where n is the normal to the habit plane and m is the shear vector. The symmetry of the cubic lattice, in the case of a fcc-to-bct transformation, results in 24 different transformation systems (n,m pairs) [42,43].

**Figure 1:**
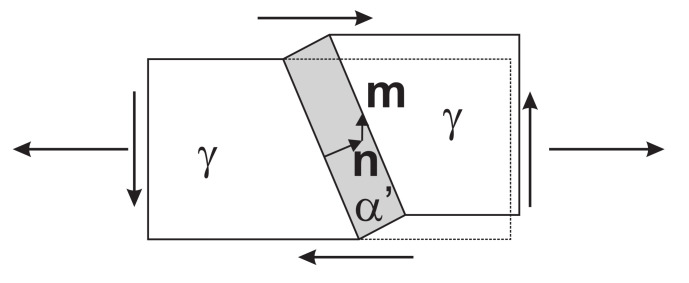
Kinematics of one martensite variant under applied stress.

When transformation evolves under the action of a stress, σ, additional mechanical driving force *U* for the transformation is supplied (Figure 1). Following [14], this can be evaluated as

(12) $$U=\sigma_{\gamma }:\left(m⊗n\right)=\sigma_{\gamma }:\frac{1}{2}\left(m⊗n+n⊗m\right)$$

where $\sigma_{\gamma }$ is the Cauchy stress in the austenite phase.

The maximum possible *U* that can be resolved in the material is an important factor to consider, since that determines the onset of transformation. Considering a polycrystalline austenite phase, each grain comprising 24 transformation systems, there will always be some grains that are optimally aligned with the applied stress to yield the maximum driving force. The maximum value of *U* can be analytically computed as

(13) $$U^{\max}=\sum_{j}\sigma_{\gamma j}\lambda_{j}$$

where $\lambda_{j}$ are the eigenvalues of the symmetric transformation deformation tensor (m⊗n+n⊗m)/2 and $\sigma_{\gamma j}$ are the eigenvalues of the local austenite stress tensor, both sorted in ascending order. $U^{\max}$ is a stress measure with units either Pa or J/m${}^{3}$.

$U^{\max}$ in Equation (13) is a generalization to an arbitrary stress state of the *U* as defined by Patel and Cohen [14]. In terms of the often-used parameters δ and γ, transformation dilatation δ=m·n and transformation shear γ=∥(1−n⊗n)·m∥, the values of λ can easily be calculated as [44]

(14) $$\lambda_{1,3}=\frac{1}{2}\left(\delta \mp \sqrt{\gamma^{2}+\delta^{2}}\right)\;,\;\lambda_{2}=0$$

The values of λ are material parameters, which are based on measured data such as transformation dilatation or crystal lattice constants. By XRD measurement, the lattice parameters of both the austenite and the resulting bcc phases can be determined. With these data, 24 transformation variants (n,m-pairs) can be calculated with respect to the austenite lattice along the procedures outlined by Wechsler et al. [42] or Bowles and MacKenzie [43]. The numerical values for all n,m-pairs for austenite containing 1.4% carbon are given in Turteltaub and Suiker [24].

When the maximum supplied driving force $U^{\max}$ exceeds a critical value, the required critical driving force $\Delta G^{\mathrm{cr}}$, according to Tamura [13], the transformation starts.

(15) $$f_{\alpha^{\prime }}>0\;\;\mathrm{when}\;\;U^{\max}>\Delta G^{\mathrm{cr}}$$

where $\Delta G^{\mathrm{cr}}$ is the lumped value of a collection of separate energy terms, such as elastic distortion of the lattice, local plastic accommodation, generation of interface, etc.

The third eigenvector of sym(m⊗n), $e_{3}$, belonging to the positive eigenvalue $\lambda_{3}$, is almost aligned with the ${\left[100\right]}_{\gamma }$ direction. This is in accordance with the observation that austenite grains, which are oriented along the [100] loading direction, are the first to transform during a tensile test. See, for example, the simulations by Turteltaub and Suiker [24] or the measurements by Jimenez-Melero et al. [45] and Hilkhuijsen et al. [46].

### 3.1. Transformation Kinetics

The amount of martensite formed is expressed as a function of $U^{\max}$.

(16) $$f_{\alpha^{\prime }}=f_{\alpha^{\prime }}^{0}+f_{\gamma }^{0}\;F\left(U^{\max}\right)$$

where $f_{\alpha^{\prime }}^{0}$ and $f_{\gamma }^{0}$ are the initial fractions of martensite and retained austenite. The function $F(U^{\max})$ describes the amount of transformation and increases monotonically from 0 to 1.

**Figure 2:**
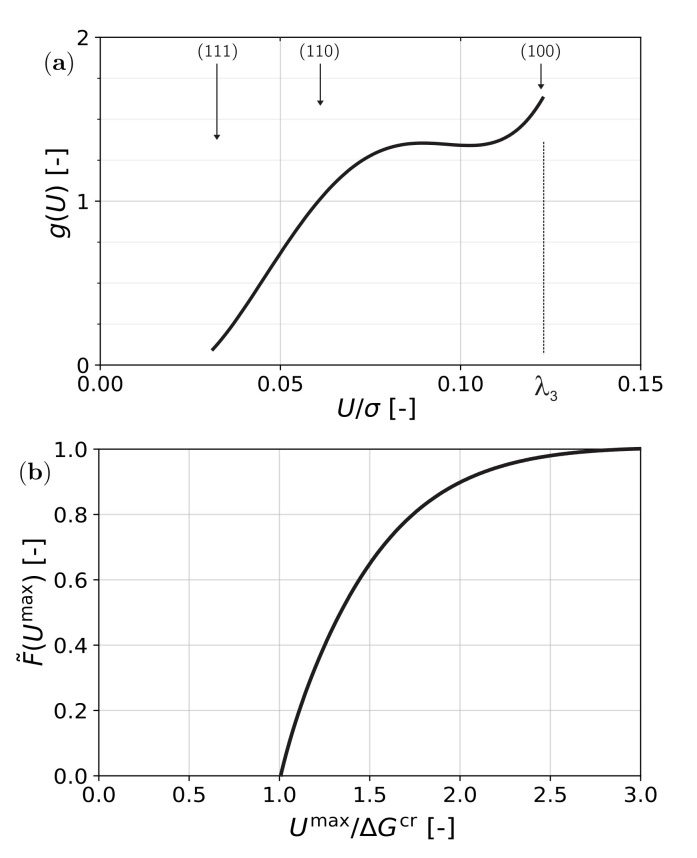
Transformation kinetics of austenitic stainless steel under uni-axial tensile stress: (**a**) distribution of driving force and (**b**) amount of transformation function $\tilde{F}\left(U\right)$.

When the texture of the material is known, a driving force distribution function can be calculated by evaluating, for each orientation, the maximum value of *U* (per unit applied stress) on any of the 24 transformation variants. In Figure 2a, the result for uni-axial tensile stress on an untextured material is shown. The function g(U/σ) is the number density of orientations with specific maximum driving force U/σ.

When a stress is applied, the function *g* is stretched along the *U*-axis to $g\left(\lambda_{3}U/U^{\max}\right)$. The amount of formed martensite is then the integral of that part of g(U) with $\Delta G^{\mathrm{cr}}<U<U^{\max}$. A formal description is obtained by considering the required critical driving force as a distribution, which is described by a Dirac δ-function centered about the value $U=\Delta G^{\mathrm{cr}}$. The cumulative distribution of grains with a mechanical driving force $U>\Delta G^{\mathrm{cr}}$ is then a Heaviside function $H(U-\Delta G^{\mathrm{cr}})$. The amount of formed martensite is then the total number of grains with a provided mechanical driving force higher than the required driving force and is evaluated as

(17) $$\tilde{F}\left(U^{\max}\right)=\frac{\lambda_{3}}{U^{\max}}\int_{0}^{U^{\max}}g\left(\frac{\lambda_{3}}{U^{\max}}x\right)H\left(x-\Delta G^{\mathrm{cr}}\right)dx$$

This is a generalization of the calculation of transformation by Tamura [13]. The result is a saturating curve as shown in Figure 2b.

### 3.2. Application to Retained Austenite

The function of Equation (17) fits remarkably well as a description of the martensitic transformation in austenitic stainless steel as was shown by Perdahcıoğlu et al. [18]. However, due to the processing route of AHSS with RA, a large variation in carbon content in individual austenite grains is to be expected, as well as a large variation in grain size of retained austenite particles. Consequently, there is also a large variation in the stability of the individual austenite grains [28,47,48]. Another aspect of variations in carbon content is that the transformation dilatation δ and transformation shear γ also vary [42,49]. Furthermore, based on the micro-mechanical simulation results obtained by Van Rompaey et al. [50], both Lani et al. [28] and Delannay et al. [29] also assumed a gradual increase in the required mechanical driving force due to the plastic hardening of the austenite.

**Figure 3:**
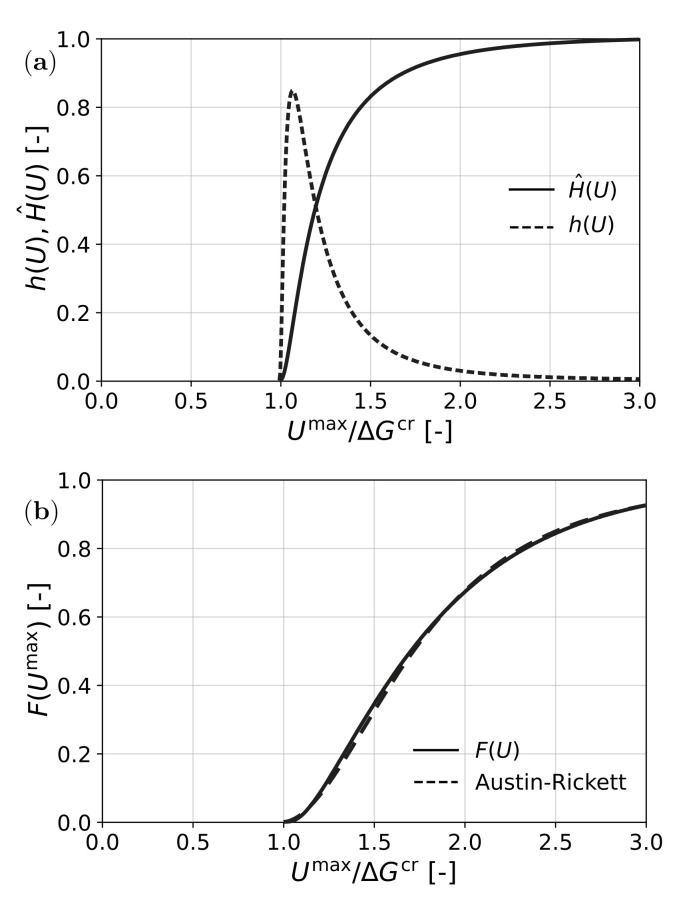
Transformation kinetics of TRIP steel: (**a**) probability density and cumulative distribution of required driving force and (**b**) function F(U) for martensite transformation.

To account for all of these effects, for the description of the transformation, instead of the Dirac function, a log normal distribution of probability density $h(U-\Delta G^{\mathrm{cr}})$ was assumed for the distribution of required $\Delta G^{\mathrm{cr}}$, as shown in Figure 3a (see, e.g., [51,52]). Then, instead of the Heaviside function, a cumulative distribution of required driving force was obtained as the function $\hat{H}\left(U-\Delta G^{\mathrm{cr}}\right)$, which is also shown in Figure 3a. The amount of formed martensite was then found as

(18) $$F\left(U^{\max}\right)=\frac{\lambda_{3}}{U^{\max}}\int_{0}^{U^{\max}}g\left(\frac{\lambda_{3}}{U^{\max}}x\right)\hat{H}\left(x-\Delta G^{\mathrm{cr}}\right)dx$$

The result is shown in Figure 3b. A suitable analytic approximation for *F* was obtained by fitting an Austin–Rickett type S-curve [53].

(19) $$F\left(U^{\max}\right)\approx 1-{\left(1+\left(p-1\right){\left(\frac{U^{\max}-\Delta G^{\mathrm{cr}}}{q\Delta G^{\mathrm{cr}}}\right)}^{r}\right)}^{\frac{1}{1-p}},\;\Leftrightarrow \;U^{\max}>\Delta G^{\mathrm{cr}}$$

which is also shown in Figure 3b. Here, *r* and *p* are parameters introduced for fitting initial rate and final saturation of *F*, while *q* determines the $U^{\max}$ for the half value of the reaction.

This result was derived for transformation in a tensile test. For other stress states, slightly differently shaped distributions *g* were obtained. The shapes of the resulting functions *F* showed very little difference with the one in Figure 3b; therefore, it was used as a generic result.

Two critical remarks about this model should be made. The first is that it only reliably predicts transformation of retained austenite, which has no pronounced texture. The incorporation of texture data into a macroscopic model such as this is still subject of study. As a second remark, it should be mentioned that, when the deformation path is not proportional, the accuracy of the model is expected to deteriorate. Under-estimation of the amount of martensite is then expected.

## 4. Transformation Plasticity

The enhancement of the formability due to presence of RA is partly attributed to the transformation plasticity. Two explanations for transformation plasticity are given in [54], enhanced yielding of the soft parent phase induced by the growth of the new phase [55] and preferential orientation of the new phase with respect to the applied stress [56]. In mechanically induced martensitic transformations, the transformation plasticity is caused by variant selection and preferential orientation of the martensite, the Magee mechanism.

The local transformation strain is defined by Equation (11). On a microscopic level, each transforming variant has its own transformation strain. In a macroscopic model, an average transformation strain would be evident, whereby the components which are not aligned with the local stress cancel out. Therefore, macroscopically, it is modeled as a dilatation plus a deviatoric component which is aligned with the deviatoric stress $S_{\gamma }$ in the austenite phase [57,58].

(20) $$D^{\mathrm{tr}}=T{\dot{f}}_{\alpha^{\prime }}=\left(\frac{1}{3}\delta 1+\frac{3}{2}T\frac{S_{\gamma }}{\sigma_{\gamma }^{\mathrm{vM}}}\right){\dot{f}}_{\alpha^{\prime }}$$

For austenitic stainless steel, a closed-form expression for the scalar factor *T* can be calculated by realizing that, for each transforming region, the following must hold:

(21) $$\sigma_{\gamma }:D^{\mathrm{tr}}=\Delta G^{\mathrm{cr}}{\dot{f}}_{\alpha^{\prime }}$$

**Figure 4:**
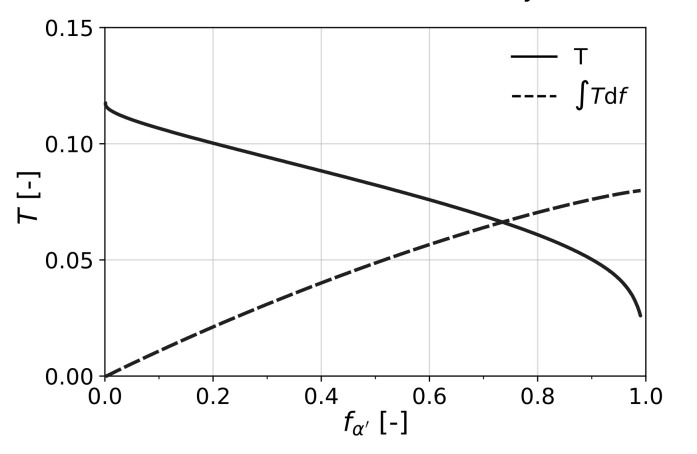
Deviatoric transformation plasticity factor *T* and its cumulative value ∫Tdf as a function of the amount of transformation $f_{\alpha^{\prime }}$.

Then, for *T*, an analytic expression can be obtained.

(22) $$T=\frac{1}{\sigma_{\gamma }^{\mathrm{vM}}}\left(\Delta G^{\mathrm{cr}}-\sigma_{\gamma }^{h}\delta \right)$$

where $\sigma_{\gamma }^{\mathrm{vM}}$ and $\sigma_{\gamma }^{h}$ are the von Mises equivalent stress and the hydrostatic stress, both in the austenite phase. This result indicates that the transformation plasticity becomes less pronounced for higher stresses, when the amount of retained austenite reduces, which can be seen in Figure 4. This can be explained by the fact that, at higher stress levels, the grains that transform are the less favorably oriented ones.

Although it is not needed for the presented model where *T* is directly computed, it is customary to define *T* as a function of the transformed volume fraction of martensite. In this case, based on Equation (22), the following linear approximation can be used:

(23) $$T\left(f_{\alpha^{\prime }}\right)\approx \left(\lambda_{3}-\frac{\delta }{3}\right)\left(1-0.55\frac{f_{\alpha^{\prime }}}{f_{\gamma }^{0}}\right)$$

For RA, again the variation in the stability of the individual austenite grains must be accounted for. The constant term $\Delta G^{\mathrm{cr}}$ in Equation (22) is replaced by the function ${\bar{\Delta G}}^{\mathrm{cr}}\left(U^{\max}\right)$, which is calculated as ($\mathrm{for}\;\;U>\Delta G^{\mathrm{cr}})$

(24) $${\bar{\Delta G}}^{\mathrm{cr}}\left(U\right)=\Delta G^{\mathrm{cr}}+\frac{\int_{0}^{U-\Delta G^{\mathrm{cr}}}xh\left(x\right)dx}{\int_{0}^{U-\Delta G^{\mathrm{cr}}}h\left(x\right)dx}$$

The result is that the total amount of transformation plasticity after the transformation has completed is only marginally changed by the variation in stability.

It was pointed out by Bhadeshia [59] that the initial amount of retained austenite in most AHSS steels is in the order of 10–20%. Therefore, the total transformation plastic strain is very low, in the order of 1–2%.

## 5. Constitutive Model

To obtain a stress–strain relation, first, the deformation rate was partitioned in an elastic rate $D^{e}$, a plastic deformation rate $D^{p}$ and a transformation plasticity rate $D^{\mathrm{tr}}$ ([30]). The elastic plus plastic rate was then partitioned among the phases.

(25) $$D_{i}=D_{i}^{e}+D_{i}^{p}=A_{i}:\left(D-D^{\mathrm{tr}}\right)$$

Straightforward differentiation of the stress, as in Equation (1), yields

(26) $$\dot{\sigma }=\sum_{i}f_{i}{\dot{\sigma }}_{i}+\sum_{i}{\dot{f}}_{i}\sigma_{i}=\sum_{i}f_{i}C_{i}:D_{i}+\sum_{i}{\dot{f}}_{i}\sigma_{i}$$

The last term on the right-hand side implies that the average stress in the already transformed martensite is transferred to the newly formed martensite as a result of transformation. On the other hand, it would be more realistic to consider that the pristine martensite has the stress of the parent austenite within which it transformed. This would make the average stress rate in martensite to be diluted as a consequence, which can be formulated as

(27) $${\dot{\sigma }}_{\alpha^{\prime }}=C_{\alpha^{\prime }}:D_{\alpha^{\prime }}+\frac{{\dot{f}}_{\alpha^{\prime }}}{f_{\alpha^{\prime }}}\left(\sigma_{\gamma }-\sigma_{\alpha^{\prime }}\right)$$

The substitution of (25) into (27) and of the result into (26) and using ${\dot{f}}_{\gamma }=-{\dot{f}}_{\alpha^{\prime }}$ yields

(28) $$\dot{\sigma }=\sum_{i}f_{i}C_{i}:D_{i}=\sum_{i}f_{i}C_{i}:A_{i}:\left(D-D^{\mathrm{tr}}\right)$$

where $C_{i}$ are the consistent elasto-plastic material tangents for phase *i*. Further details of the constitutive model and its implementation are given in [23].

## 6. Comparison with Experimental Data

Lani et al. [28] presented extensive stress and strain measurements on a specific TRIP steel with a chemical composition of 0.29 wt.% C, 1.42 wt.% Mn, 1.41 wt.% Si. The strains of the individual phases were measured by digital image correlation on SEM micrographs acquired in situ during tensile tests. Similarly, the stresses in the individual phases were determined by neutron diffraction using the elastic strains of the fcc phase austenite and the combined bcc phases, ferrite, bainite and martensite. Stresses in individual bcc phases cannot be obtained since these give identical diffraction peaks. These results were combined to obtain the stress and strain partitioning among the phases.

The mechanical response of this TRIP steel for uniaxial deformation was simulated using the model presented above. All available material data in [28] were used directly for each phase and are given in Table 1. The data for the bainite phase were estimated based on the data provided for the combined ferrite–bainite data. All phases were elastic–plastic with isotropic hardening according to a Swift rule.

(29) $$\sigma^{y}\left(\varepsilon^{p}\right)=\sigma^{y_{0}}{\left(1+k\varepsilon^{p}\right)}^{n}$$

where $\sigma^{y_{0}}$ and $\varepsilon^{p}$ are the yield stress and the equivalent plastic strain, respectively.

The data used as phase transformation parameters in the model are given in Table 2. The transformation shear and dilatation were taken from [24], the required critical driving force $\Delta G^{\mathrm{cr}}$ was chosen to fit the experimental results from [28], although Jacques et al. [60] showed that accurate measurement of retained austenite is not trivial.

**Figure 5:**
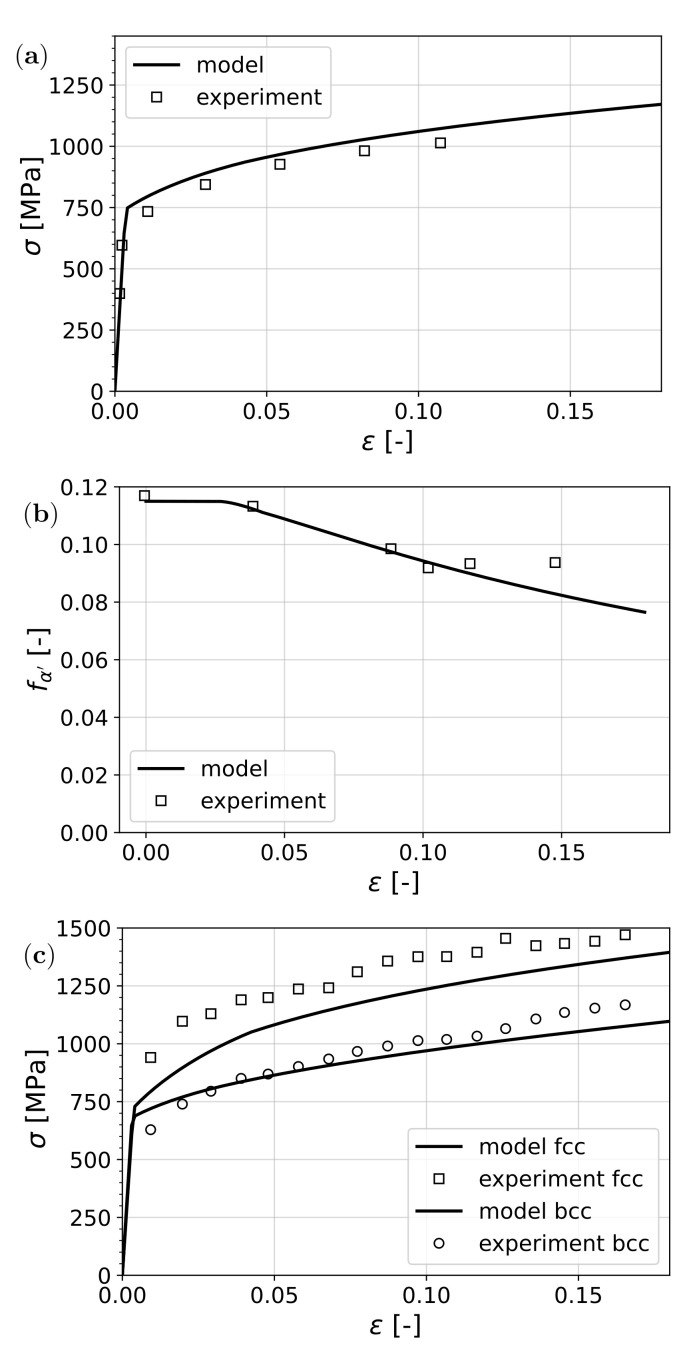
Prediction of the model compared to experimental results by Lani et al. [28]: (**a**) stress vs. strain, (**b**) retained austenite vs. strain and (**c**) stress resolved in bcc and fcc phases vs. overall strain.

In Figure 5, the results of the comparison of the model and the measurements from [28] are shown. It can be seen that the overall uniaxial stress–strain curve, as well as the transformation of austenite, was captured satisfactorily. The prediction of the stress partitioning among the phases also fits reasonably well with the measurements.

Another thorough experimental study has been performed on TRIP-assisted dual-phase steels by Ennis et al. [61]. The stress–strain response and the transformation were recorded with high accuracy, especially in the early stages of plastic deformation. Furthermore, thanks to the high-energy XRD technique, the stress partitioning into different phases during deformation (in situ) was captured. In this steel, it has been observed that the transformation of retained austenite starts before the onset of plastic deformation of the softest phase, ferrite; hence, a clear plastic yield point identification is not possible. Based on all data provided in the article with respect to the yield stress of individual phases, the mechanical parameters in Table 3 were used. The hardening parameters, as well as the yield stress of martensite, are not reported in the article; therefore, they were determined based on fitting the resulting tensile curve to the experiment. Transformation parameters for this material were calibrated as given in Table 4.

**Figure 6:**
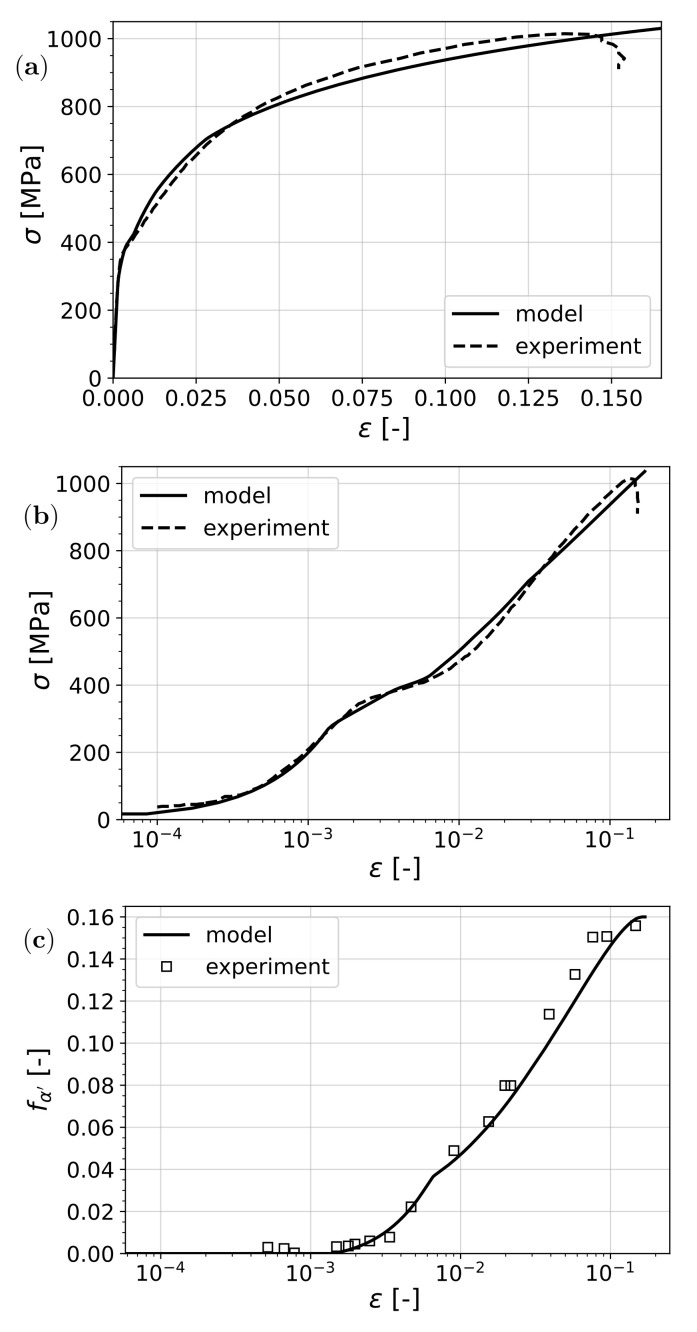
Prediction of the model compared to experimental results by Ennis et al. [61]: (**a**) stress vs. strain, (**b**) stress vs. log strain, and (**c**) transformed martensite vs. log strain.

The results of the comparison are shown in Figure 6. Overall, a good fit in terms of both the predicted stress–strain response and the transformation was observed.

On the experimental (log-scale) stress–strain curve, some interesting points can be identified by making use of the model results where the discontinuities in the tangent are more pronounced. The first bend corresponds to the start of transformation as it happens before the material reaches the yield stress of ferrite, which is the softest phase. At the yield point of ferrite, another slope change can be observed. When austenite yields, the transformation curve undergoes an abrupt change in slope. This is due to the fact that the stress in austenite is the main driving factor for transformation and, when this phase yields, the strain partitioning changes abruptly, causing an apparent change in slope with the overall strain.

Both of these studies provided very valuable quantitative information on the mechanical properties of phases where direct measurement is possible. These data, as much as possible, were used directly in the current model. Therefore, the goal here was not the fitting of these parameters in order to obtain quantitatively the *best* fit but rather to demonstrate the predictive capability of the model based on measured data that describe the physics of the process. By improving the reliability of the measured data, a more quantitative evaluation of the accuracy of the proposed model can be made.

On the other hand, direct measurement of phase properties in multi-phase steels and especially those that undergo phase transformation remains difficult. An immediate solution would be isolating phases with the exact chemical composition found in the mixture and manufacturing macroscopic test samples from these. However, this requires precise control on the production process of steel which, in practice, is limited to very-small-scale samples and not actual production grade steels. An additional complication to this is the fact that the phases found in these materials usually have a distribution of chemical composition, as well as residual stresses and initial plastic deformation, associated with the production process. Micro- and nano-indentation methods provide valuable information about phase properties, while having difficulties with the very local nature of the measurements, as the effects of the surrounding microstructure are difficult to quantify [62]. This, in turn, requires inverse analysis requiring averaging methods in order to estimate the properties of each individual phase [26].

## 7. Effect of Transformation on Stability of Sheet Metal

In sheet metal forming of AHSS, one of the limiting criteria for forming is the cross-sectional stability of the material, in other words, local necking. It is known that the stability of the sheet depends on the in-plane loading conditions; therefore, commonly, for each grade, a forming limit curve (FLC) is determined. The goal in this section is to show the effect of the transformation of retained austenite on the FLC of a certain grade of steel. The choice for the application is the same steel used in the study by Ennis et al. [61].

For opposite signed in-plane major and minor strains, i.e., second quadrant, it is well established that the maximum tension criterion by Hill predicts the onset of local necking reliably. However, within the first quadrant, for positive minor and major strains, another approach, proposed by Marciniak et al. [63] (Marciniak–Kuczynski, M-K), proved to be reliable. The following procedures are used in finding the onset of instability.

The stress response of the material was computed using an incremental strain-driven approach. Therefore, for each increment, the plane-stress condition was enforced using a nonlinear solution algorithm based on Newton–Raphson iterations. In these iterations, a solution to the non-prescribed strain components was sought that makes all out-of-plane stress components vanish. For each converged incremental result, the following conditions were checked in order to determine the onset of instability.

*Maximum tension criterion*

It was postulated that cross-sectional instability starts when the tension reaches a maximum value. This results in the following formulation [64]:

(30) $$\sigma_{11}^{\left(t\right)}\left(1+\Delta \varepsilon_{33}\right)-\sigma_{11}^{\left(t-1\right)}\leq 0$$

where *t* is the current time increment and $\Delta \varepsilon_{33}$ is the incremental thickness strain.

*M-K criterion*

As opposed to maximum tension, in order to evaluate the M-K criterion, the history of deformation must be known. An imaginary section of the material was considered with a thickness imperfection that created a stress difference for the same strain path. For more detailed treatment of the implementation of the M-K criterion, see, for instance [64].

The evolution of the thickness imperfection is defined as

(31) $$\omega_{t}=\omega_{0}\;\exp\left(\varepsilon_{33}^{A}-\varepsilon_{33}^{B}\right)$$

where A and B denote the homogeneous and imperfect regions of the material, respectively—$\omega =t^{A}/t^{B}$ and $\omega_{0}=t_{0}^{A}/t_{0}^{B}$.

Considering force equilibrium and strain compatibility, the following conditions arose:

(32) $$\sigma_{11}^{B}=\omega \;\sigma_{11}^{A},\;\Delta \varepsilon_{22}^{A}=\Delta \varepsilon_{22}^{B}.$$

Then, within the imperfection, the unknown strain and stress components were solved using the Newton–Raphson method. The onset of instability was found when

(33) $${\left(\Delta \varepsilon_{33}^{p}\right)}^{B}/\Delta \varepsilon_{33}^{A}\geq \xi .$$

In obtaining the results, the following values for the imperfection and the strain ratio were used:

(34) $$\omega_{0}=1/0.9989,\;\xi =50.$$

*Results*

**Figure 7:**
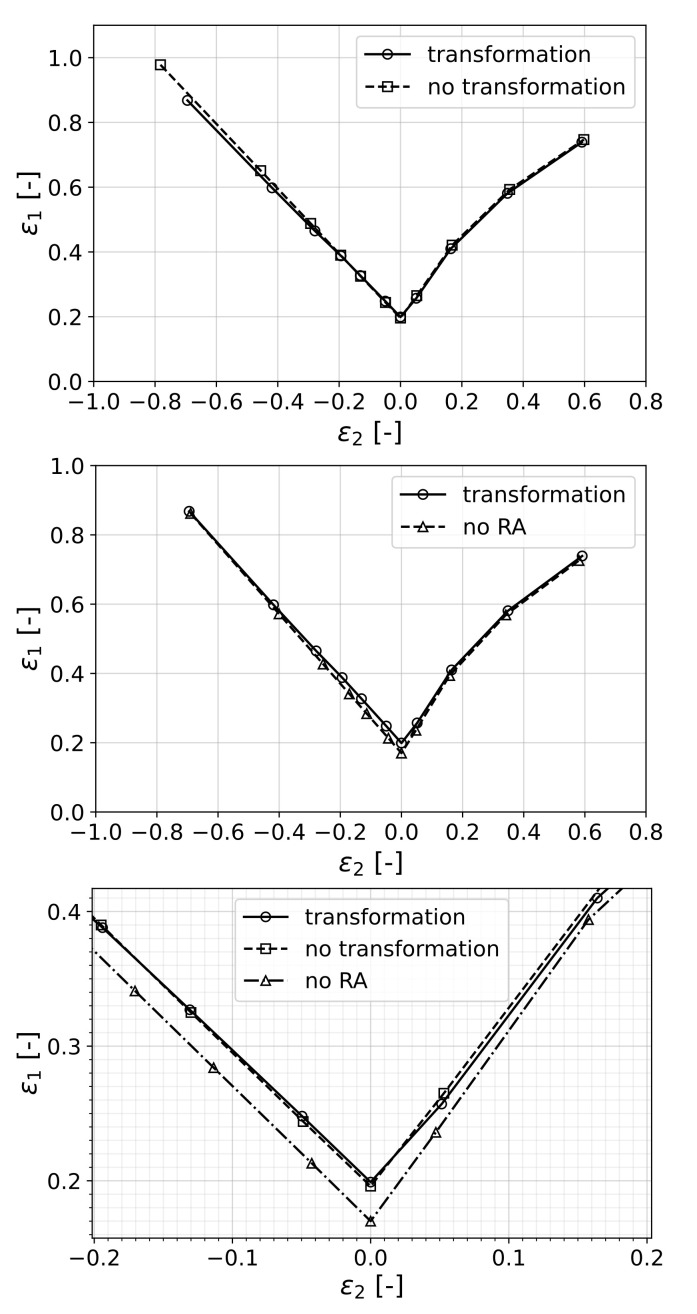
Prediction of the model in terms of onset of localized necking where *no transformation* represents the case with a very stable RA and *no RA* is the case where RA was replaced with initial martensite.

The resulting forming limit curves for both transforming and non-transforming materials are shown in Figure 7. For this analysis, the material properties that were fitted to the experiments of [61] were used.

**Figure 8:**
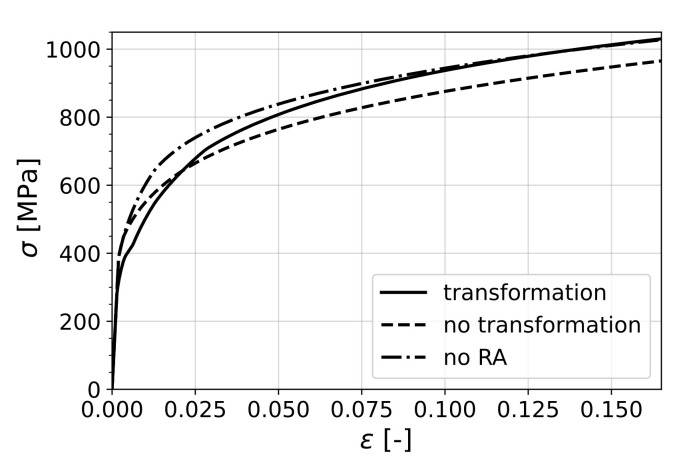
Simulation of uniaxial tension with and without transformation, where *no transformation* represents the case with a very stable RA and *no RA* is the case where RA was replaced with initial martensite.

In order to isolate and single out the effects of mechanically induced phase transformation on the formability of the material, two cases were chosen. In the first one, denoted as *no transformation*, the material had exactly the same phase fractions and phase properties as the original one but transformation was suppressed by assigning a high transformation energy barrier ($\Delta G^{\mathrm{cr}}$). In the second case, the RA phase in the original material was replaced by initial martensite (keeping the mechanical properties of the phase unchanged) in order to represent a pure dual-phase steel, denoted by *no RA*. The behavior of these individual cases under uniaxial tension are shown in Figure 8.

It can be seen that the existence of the retained austenite phase was, in each case, transforming or non-transforming, beneficial in terms of formability, as the material without RA performed the worst. However, as the stress state approaches shear in the second quadrant and equibiaxial tension in the first, the difference between the curves vanishes.

In order to understand this result, it is worth considering Equation (30) as the criterion that determines the onset of localization for uniaxial loading. This equation can be interpreted as the competition between the change of cross-sectional tension due to the hardening of the material and the change that comes as a result of scaling of the current tension with the reduction in thickness. In the case of transformation of retained austenite, there was an added ductility to the material in terms of transformation strain, which was around 1.3% (see Figure 4). On the other hand, the increase in flow stress due to transformation to the harder martensite phase acted as a negative factor towards formability, considering the criterion mentioned above.

Hence, in the case of non-transforming RA, the material lost the added ductility from transformation; however, since the flow stress was also reduced, it retained a high formability. On the other hand, the material without RA had high flow stress without the added ductility due to TRIP; therefore, it performed the worst. Therefore, it is clear that the material with transformation of RA benefited from transformation plasticity while keeping the high strength due to a complete transformation to martensite.

## 8. Conclusions

The behavior of multi-phase steels and especially those that comprise the retained austenite phase was modeled using a combination of the mean-field modeling approach and a physics-based, mechanically induced transformation model.

It is shown that the self-consistent analytical homogenization scheme could be used successfully for modeling multi-phase steels, which is supported by experimental validation of the stress partitioning into different phases.

It was observed experimentally that, in the case of retained austenite, complete transformation was not always reached. This is taken into account in the proposed model by considering a distribution of the energetic transformation barrier due to a distribution of carbon and other factors in retained austenite islands. This, in turn, was convoluted with the resolved driving force distribution to obtain the overall transformation curve as a function of the maximum resolved driving force, which gave a good prediction in terms of transformation validated by experiments in the literature.

Mechanically induced transformation of retained austenite was modeled as a stress–driven process. In fitting with experimental results, it could be seen that this approach proved to be valid, as transformation started before any significant slip activity was expected in the retained austenite phase. In addition, the theoretically predicted transformation strain was validated with the experimental results found in the literature quantitatively.

The effects of transformation of retained austenite on the cross-sectional stability of sheet metals is an open question. The numerical study carried out in terms of forming limit curve prediction answered this question by showing that, while the added ductility due to transformation plasticity was beneficial to formability, the associated increase in flow stress had a negative effect. However, compared to a material without RA, formability was enhanced.

These findings clearly depend on the assumed phase properties, especially for the transformed martensite phase, which has an influence on the hardening slope as well as the flow stress. The determination of correct parameters for each phase however remains a big experimental challenge.

## Figures and Tables

**Table 1:** Mechanical properties of each phase used in the simulation of TRIP steel.

Phase	Fraction	*E* (GPa)	ν	$\sigma^{y_{0}}$ (MPa)	*k*	*n*
austenite	0.115 *	187	0.3	1130	80	0.20
martensite	0.0 *	187	0.3	2000	800	0.05
ferrite	0.555	220	0.3	645	50	0.175
bainite	0.330	220	0.3	805	50	0.175

* initial.

**Table 2:** Data for transformation of retained austenite.

Transformation dilatation	δ	0.039	(-)
Transformation shear	γ	0.181	(-)
Required critical driving force	$\Delta G^{\mathrm{cr}}$	110	(MPa)
Transformation parameter	*p*	1.5	(-)
Transformation parameter	*q*	0.8	(-)
Transformation parameter	*r*	1.8	(-)

**Table 3:** Mechanical properties of each phase used in the simulation of TRIP-aided DP steel.

Phase	Fraction	*E* (GPa)	ν	$\sigma^{y_{0}}$ (MPa)	*k*	*n*
austenite	0.16 *	200	0.3	493	110	0.20
(transformed) martensite	0.0 *	200	0.3	1265	800	0.08
ferrite	0.78	200	0.3	389	500	0.205
(original) martensite	0.06	200	0.3	865	800	0.08

* initial.

**Table 4:** Data for transformation of retained austenite in TRIP-aided DP steel.

Transformation dilatation	δ	0.039	(-)
Transformation shear	γ	0.181	(-)
Required critical driving force	$\Delta G^{\mathrm{cr}}$	30	(MPa)
Transformation parameter	*p*	1.8	(-)
Transformation parameter	*q*	0.4	(-)
Transformation parameter	*r*	1.8	(-)

## Data Availability

Data sharing not applicable.
